# Microbubble protein delivery for Parkinson’s

**DOI:** 10.1186/2050-5736-3-S1-O21

**Published:** 2015-06-30

**Authors:** James Keenan, Umar Iqbal, Maria Moreno, Jagdeep Sandhu

**Affiliations:** 1 Artenga Inc., Ottawa, Ontario, Canada; 2 National Research Council of Canada, Ottawa, Ontario, Canada

## Background/introduction

Microbubbles (MBs) are small (1-5 μm diameter), perfluorcarbon gas filled lipid microspheres used for contrast enhancement during ultrasound imaging. MBs (Definity^®^, others) are approved for contrast echocardiography and widely used (6 M patient injections) as ultrasound contrast agents with established pharmacokinetics and clearance data. Recent research has shifted to the therapeutic potential of MBs and the NRC and Artenga Inc. developed a novel strategy (Patent to be filed) to load biologics onto MBs with high, consistent loading using a novel clinically scalable methodology.

Investigators at Sunnybrook Research Institute have pioneered MR guided focused ultrasound (MRgFUS) plus MB techniques to deliver a wide range of therapeutic agents through the blood-brain-barrier (BBB) and developed a state of the art MRgFUS system for animal models. MRgFUS can precisely target the substantia nigra (SN) and striatum (ST) for simultaneous targeting of multiple brain regions.

Neurotrophic factors (NTFs) are being developed in the hope of halting or reversing the progression of neuronal loss in chronic neurodegenerative diseases, however, their clinical utility is limited by drug delivery issues. Recently the University of Helsinki discovered a novel NTF, cerebral dopamine neurotrophic factor (CDNF), with a unique structure and mode of action. CDNF has demonstrated improved efficacy compared to GDNF in various preclinical models of PD. Artenga, Inc., Sunnybrook Research Institute, the National Research Council of Canada (NRC), and the University of Helsinki plan is to use MR guided focused ultrasound (MRgFUS) and neurotrophic drug-loaded microbubbles (NTF-MBs) for targeted, noninvasive, blood brain barrier (BBB) drug delivery of CDNF.

## Methods

Although CDNF neuroprotection and neurorestoration data are promising, clinical development might be hampered by its inability to cross the BBB and rely on delivery methods that are either highly invasive or non-targeted. Hence, our focus on non-invasive targeted delivery of CDNF into the SN and ST using CDNF-MBs coupled with MRgFUS to open the BBB for the delivery of therapeutic doses of CDNF. Artenga’s goal is to commercialize our technology to treat multiple diseases with different compounds and tumour-targeting agents. The Artenga-NRC MB conjugation technology permits extremely high and consistent drug loading per MB for a wide range of compounds including antibodies, proteins, complex 3D folded antibodies, antibody drug conjugates, and gene therapy. The covalent bond results in reliable *in vivo* performance. The technology can also be adopted for cancer treatments. A novel therapeutic method of action was developed at Sunnybrook Research Institute to damage tumour vasculature using focused ultrasound and MBs. The technique has shown synergy with chemotherapy to significantly increase tumour reduction compared to the drug alone. Testing is ongoing with tumour targeting MBs. Indications suitable for treatment with drug loaded MBs and focused ultrasound include Parkinson’s disease, other CNS disorders, and different types of cancer. For cancer applications a tumour targeting, drug loaded MB will be used.

